# A Case of Anaphylactic Shock Suspected to Be Caused by Bovine Serum Albumin Tissue Adhesive (BioGlue) During Thoracoabdominal Aortic Replacement

**DOI:** 10.7759/cureus.62678

**Published:** 2024-06-19

**Authors:** Rintaro Kinjo, Takenori Kojima, Tomoya Miyamoto, Takeshi Sakaguchi, Ryusuke Suzuki

**Affiliations:** 1 Cardiovascular Surgery, Japanese Red Cross Kumamoto Hospital, Kumamoto, JPN

**Keywords:** bioglue, surgical adhesive, thoracoabdominal aortic aneurysm repair, thoracoabdominal aortic aneurysm, intraoperative shock, anaphylaxis, perioperative anaphylaxis

## Abstract

Bovine serum albumin (BSA) tissue adhesive (BioGlue, Cryolife Inc., Kennesaw, GA, USA) is often used as a hemostatic adjunct in adult patients undergoing open surgical repair of large vessels, such as the aorta, femoral, and carotid arteries. We present a case of intraoperative anaphylaxis attributed to the use of BioGlue during thoracoabdominal aortic replacement. The patient suddenly developed hypotension, hypoxemia, and airway edema after the BioGlue application. BioGlue is composed of purified BSA and glutaraldehyde. Despite the BSA component being an allergen that can cause anaphylaxis in some patients, reported occurrences of anaphylaxis are extremely rare. We need to recognize BSA tissue adhesive as a potential allergen. In the event of an intraoperative shock, prompt recognition and investigation of the underlying cause are necessary.

## Introduction

In cardiovascular surgery, bovine serum albumin (BSA) tissue adhesive (BioGlue) is used as a hemostatic adjunct in adult patients undergoing open surgical repair of large vessels, such as the aorta, femoral, and carotid arteries, in conjunction with standard techniques such as sutures and staples. It is composed of purified BSA and glutaraldehyde. The Food and Drug Administration provides cautionary notes regarding anaphylaxis [1]. Based on hypersensitivity tests, the risk of anaphylactic reaction by repeated use or long-term exposure to BioGlue is low. Still, once sensitized, other medical devices or medicines containing BSA can induce an anaphylactic reaction [1].

There have been a few reported cases of intraoperative anaphylactic shock occurring shortly after the initial use of BioGlue, with implications suggesting an association with pork-cat syndrome [2,3]. Pork-cat syndrome is a rare allergy condition in which patients allergic to cat dander also develop allergic reactions to pork and other mammalian meat products, such as BSA, due to cross-reactivity with serum albumin [2]. However, there have been few reported instances of intraoperative anaphylactic shock following the second use of BioGlue.

Here, we present a rare case of intraoperative anaphylaxis attributed to BioGlue during thoracoabdominal aortic replacement. This case underscores the importance of considering uncommon etiologies in the perioperative period and highlights the challenges in effectively managing such cases.

## Case presentation

**Figure 1:**
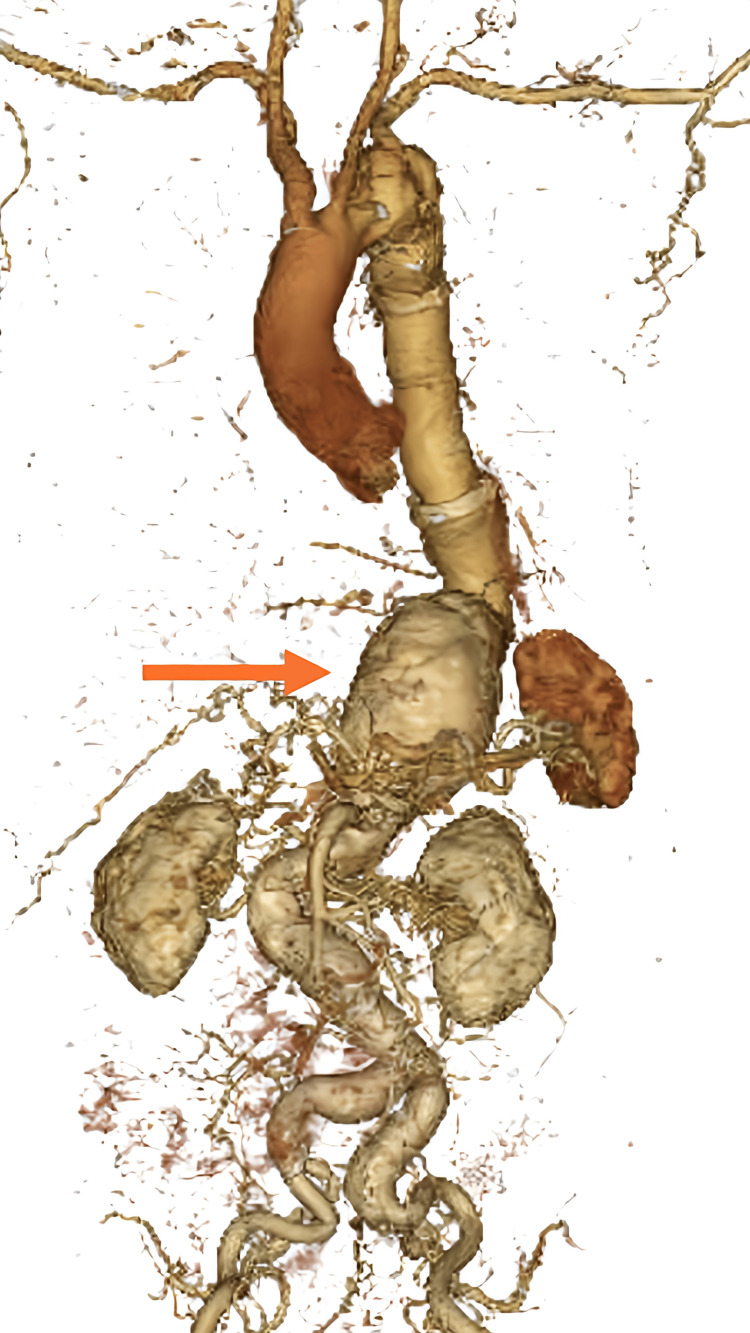
Contrast-enhanced CT scan reconstructed in 3D before admission A contrast-enhanced CT scan shows the thoracoabdominal aortic aneurysm. The diameter at the distal anastomosis of the artificial graft from the previous surgery is 45 mm, with a waist of 30 mm just above the diaphragm and 60 mm at the level of the diaphragm (orange arrow). The aneurysm is fusiform and converges at the superior mesenteric artery branching point.

The patient is a 76-year-old male with medical comorbidities of type 2 diabetes, dyslipidemia, and a surgical history of ruptured descending aortic aneurysm requiring emergent open thoracic aortic replacement four years before. In this surgery, the aneurysm was replaced with an artificial graft (Triplex, Termo Corp., Tokyo, Japan). He was also diagnosed with a thoracoabdominal aneurysm distal to the replacement segment, which had a maximum diameter of 45 mm. Surveillance imaging studies demonstrated interval enlargement to >60 mm (Figure 1). Given the interval growth, the patient was evaluated for thoracoabdominal aortic aneurysm repair. On the contrast-enhanced CT scan just before admission, the diameter of the anastomosis of the artificial graft from the previous surgery was 45 mm in diameter, with a waist of 30 mm just above the diaphragm and 60 mm at the level of the diaphragm (Figure 1). The aneurysm was fusiform and converged at the superior mesenteric artery branching point. The patient did not report any symptoms. He had only a pollen allergy, with no known allergies to food or medication. No allergy tests, such as for BSA, were conducted prior to surgery.

**Figure 2:**
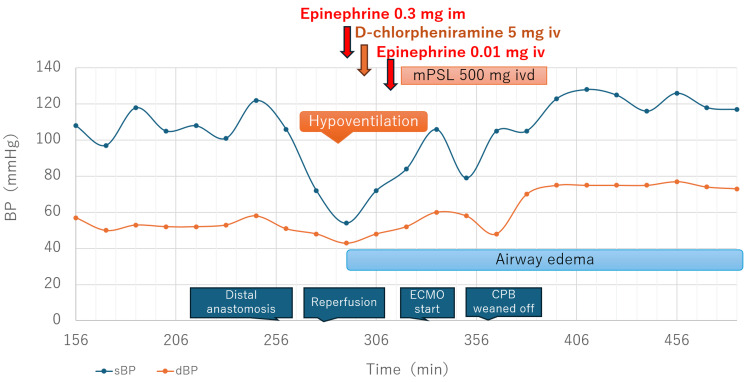
Time course of surgery, BP, and administered medications BP, blood pressure; sBP, systolic BP; dBP, diastolic BP; im, intramuscular injection; iv, intravenous injection; ivd, intravenous drip; mPSL, methylprednisolone; CPB, cardiopulmonary bypass

During the operation, a new artificial graft (Triplex) was anastomosed to the thoracic aorta proximal to the aneurysm, and BioGlue was applied. Subsequently, the aortic aneurysm was incised, and the distal abdominal aorta was sutured and anastomosed, followed by the application of BioGlue. After reperfusion, the patient developed shock with hypotension and hypoxemia, accompanied by a marked decrease in vascular volume and significant airway edema (Figure 2). No bleeding was observed in the thoracic or abdominal cavities or retroperitoneum, and no findings suggestive of acute coronary syndrome or pulmonary embolism were observed. The anaphylactic shock was suspected, and adrenaline was administered intramuscularly (0.3 mg) and intravenously (0.01 mg) along with intravenous (IV) d-chlorpheniramine (5 mg), and an IV drip infusion of methylprednisolone (500 mg) was initiated. Although blood pressure and ventilation improved, airway edema persisted, and the ventilation volume could not be maintained. Therefore, veno-arterial extracorporeal membrane oxygenation (VA-ECMO) was initiated, and the patient was weaned off cardiopulmonary bypass. The patient was admitted to the ICU after surgery.

The patient regained consciousness on the same day, and no signs of paralysis were confirmed. Airway edema improved the day after surgery, and respiratory status was stable, leading to ECMO removal. Subsequently, the patient developed dehydration and oliguria, progressing to acute kidney injury, requiring continuous hemodiafiltration (CHDF) from postoperative day 4. After volume reduction, the patient was extubated and switched from CHDF to intermittent hemodialysis on postoperative day 7. Dialysis was discontinued on postoperative day 10. Additionally, the post-operational course was complicated by acute pancreatitis, which was detected on CT imaging on postoperative day 10. This prevented sufficient rehabilitation, leading to transfer to another facility for rehabilitation on postoperative day 36.

## Discussion

Several factors contribute to the development of shock in the operating room. Shock results from four potential pathophysiological mechanisms that are not necessarily exclusive [4]. These mechanisms include hypovolemia resulting from internal or external fluid loss; cardiogenic factors such as acute myocardial infarction or cardiac arrhythmias; obstruction such as pulmonary embolism, cardiac tamponade, or tension pneumothorax; and distributive factors such as severe sepsis or anaphylaxis due to the release of inflammatory mediators. Identifying the underlying cause of shock is essential to providing appropriate treatment. 

In this case, the sudden onset of hypoventilation and hypotension occurred during surgery. We recognized that the patient was in shock and searched for the cause. First, there was no evidence of thoracic, intra-abdominal, or retroperitoneal hemorrhage, thus making hypovolemic shock less likely. There were no electrocardiographic changes or left ventricular wall hypokinesia on transesophageal echocardiography (TEE) suggestive of acute myocardial infarction leading to cardiogenic shock. No echocardiographic findings suggested cardiac tamponade or tension pneumothorax, which could cause obstructive shock. Distributive (anaphylactic) shock was suspected because of marked facial edema and redness, airway edema during bronchoscopy, and circulatory collapse with decreased left ventricular volume on TEE.

Epinephrine, d-chlorpheniramine, methylprednisolone, and saline were administered in this case. In case of refractory hypotension, vasopressin, norepinephrine, and methylene blue could also be considered. In addition, inhaled beta-2 agonists such as albuterol or salbutamol can be used to treat severe bronchospasm [5,6]. 

The collapse occurred in the middle of the anastomosis procedures, which could not be stopped. There are opinions that surgery should be stopped if anaphylaxis occurs at a stage where it can be safely halted. However, previous studies suggest that there were no significant differences in major hypersensitivity-related complications for cases with Ring and Messmer Grade III reactions (cardiovascular collapse, tachycardia, and cutaneous features), regardless of whether surgery had been abandoned or continued once resuscitation had been achieved [7]. This can also be said for cardiac surgery cases [8]. 

At this time, airway edema persisted, and VA-ECMO was initiated. There have been multiple case reports on using ECMO for refractory anaphylaxis [9-11]. In cases where adequate ventilation is difficult owing to airway edema and bronchospasm caused by anaphylaxis and hemodynamics are unstable, the introduction of ECMO can be an effective measure [12].

There were no new medication administrations just prior to deterioration, and the timing coincided with the resumption of circulation in the lower body after artificial graft anastomosis, suggesting that either BioGlue or the artificial graft could cause anaphylaxis. Between the two, BioGlue appeared more suspicious as the cause. If the graft were the cause, the condition would not have improved because the patient had been constantly exposed to blood flow. BioGlue is composed of purified BSA and glutaraldehyde, which was used in the surgery four years prior to this event, raising the suspicion of prior sensitization. Protamine, which can cause shock during heart surgery, was administered approximately 90 minutes after the crush, just after weaning off from CPB. Therefore, due to the timeline not aligning, we did not suspect protamine reaction as the cause.

A case of a 67-year-old man with a history of atopy who underwent a Bentall procedure for a thoracic aortic aneurysm has been reported. After using BioGlue, the patient experienced bronchospasm and hypotension [2]. In another case, a 53-year-old man underwent artificial vascular replacement surgery for an abdominal aortic aneurysm using BioGlue. Subsequently, the patient experienced bronchospasms and hypotension [3]. Both patients had a history of owning a cat and rhinitis. Sensitization to cat serum albumin has been suggested to be associated with the “pork-cat syndrome,” in which ingestion of meats such as pork, beef, and lamb can trigger allergic reactions. In our case, although no diagnostic allergy test was conducted and no history of reaction after consumption of beef and pork, the patient had a long history of owning cats, indicating potential sensitization to cat proteins and a minor possibility of anaphylactic shock due to cross-reactivity. Specific IgE tests for BSA and cat serum albumin and serum tryptase level could have made a significant contribution to clarifying the pathophysiology.

According to a meta-analysis, the overall in-hospital mortality rate for open repair of thoracoabdominal aortic aneurysms was 11.26%. In terms of morbidity, the cardiac event rate was 4.41%, the stroke rate was 3.11%, the acute kidney injury rate was 11.65%, and the spinal cord ischemia rate was 8.26% [13]. Those are expected mortality and morbidity rates for this case, but the actual course was affected by anaphylactic shock. Perioperative anaphylactic shock is potentially life-threatening. A study in the USA found that the proportion of fatal outcomes after perioperative anaphylaxis was 2%, and its risk factor included cardiac procedures [14]. It also has significant impacts on the postoperative course and outcomes. A single-center retrospective study showed that the mean postoperative length of stay for open thoracoabdominal aortic aneurysm repair was seven days [15]. However, the duration can be extended due to complications, such as renal failure, spinal cord deficits, and pulmonary complications [16]. In this case, the patient was under severe hypotension and hypoxia during the anaphylactic episode, which could result in end-organ ischemia and injury, especially the kidney. Airway edema and bronchospasm from anaphylaxis could lead to postoperative ARDS. Moreover, excessive intraoperative fluid administration and renal injury led to pulmonary edema, resulting in prolonged intubation periods and ICU stays. Additionally, postoperative pancreatitis contributed to extended hospital stays and delayed rehabilitation, resulting in a total of 32 days of hospitalization, which was longer than expected. Thus, perioperative anaphylaxis worsened the outcome.

## Conclusions

Here, we report a case of intraoperative anaphylactic shock temporally associated with BioGlue. During surgery, if unexplained circulatory collapse, bronchospasm, or airway edema is observed, it is crucial to promptly diagnose and treat anaphylactic shock after excluding causes such as bleeding to save the patient’s life. Patients with no known history of allergies to meat or cats may experience anaphylaxis after a second use of BioGlue. Anaphylactic shock makes patients’ prognoses worse. Patients previously exposed to BioGlue may develop a hypersensitivity reaction when using it for the second time, and the surgical team must be aware of this possibility and be ready to respond.

## References

[1] (2024). Food and Drug Administration: Summary of safety and effectiveness CryoLife, Inc., BioGlue® Surgical Adhesive. https://www.accessdata.fda.gov/cdrh_docs/pdf/p010003b.pdf.

[2] Hilger C, Clark E, Swiontek K, Chiriac AM, Caimmi DP, Demoly P, Bourrain JL (2020). Anaphylaxis to bovine serum albumin tissue adhesive in a non-meat-allergic patient. J Investig Allergol Clin Immunol.

[3] Dewachter P, Jacquenet S, Beloucif S, Goarin JP, Koskas F, Mouton-Faivre C (2019). Pork-cat syndrome revealed after surgery: Anaphylaxis to bovine serum albumin tissue adhesive. J Allergy Clin Immunol Pract.

[4] Vincent JL, De Backer D (2013). Circulatory shock. N Engl J Med.

[5] Tacquard C, Iba T, Levy JH (2023). Perioperative anaphylaxis. Anesthesiology.

[6] Dewachter P, Savic L (2019). Perioperative anaphylaxis: pathophysiology, clinical presentation and management. BJA Educ.

[7] Sadleir PH, Clarke RC, Bozic B, Platt PR (2018). Consequences of proceeding with surgery after resuscitation from intra-operative anaphylaxis. Anaesthesia.

[8] Norawat R, Vohra A, Parkes A, O'Keeffe NJ, Anipindi S, Maybauer MO (2022). Incidence and outcome of anaphylaxis in cardiac surgical patients. Ann Card Anaesth.

[9] Yu HK, Park M, Lee SH, Woo JW, Kang DH, Byun JH, Ok SH (2022). Early use of ECMO for refractory Kounis syndrome concealed by general anesthesia-a case report. Medicina (Kaunas).

[10] Grafeneder J, Ettl F, Warenits AM (2022). Multi-phasic life-threatening anaphylaxis refractory to epinephrine managed by extracorporeal membrane oxygenation (ECMO): a case report. Front Allergy.

[11] Amano Y, Matsuura A, Tamura T, Kato Y, Kameyama N, Takazawa T, Nishiwaki K (2023). Life-threatening chlorhexidine anaphylaxis caused by skin preparation before chlorhexidine-free central venous catheter insertion: a case report and literature review. J Anesth.

[12] Scaravilli V, Grasselli G, Benini A (2017). ECMO for intractable status asthmaticus following atracurium. J Artif Organs.

[13] Moulakakis KG, Karaolanis G, Antonopoulos CN (2018). Open repair of thoracoabdominal aortic aneurysms in experienced centers. J Vasc Surg.

[14] Gonzalez-Estrada A, Campbell RL, Carrillo-Martin I, Renew JR, Rank MA, Volcheck GW (2021). Incidence and risk factors for near-fatal and fatal outcomes after perioperative and periprocedural anaphylaxis in the USA, 2005-2014. Br J Anaesth.

[15] Wang S, Wang C, Gao Y, Tian Y, Liu J, Wang Y (2024). Risk factors of 30-day and long-term mortality and outcomes in open repair of thoracoabdominal aortic aneurysm. J Cardiothorac Surg.

[16] Huynh TT, Miller CC 3rd, Estrera AL, Sheinbaum R, Allen SJ, Safi HJ (2002). Determinants of hospital length of stay after thoracoabdominal aortic aneurysm repair. J Vasc Surg.

